# Combustion-Synthesized BaAl_2_O_4_: Eu^2+^, Nd^3+^, Pr^3+^ Triple-Co-Doped Long-Afterglow Phosphors: Luminescence and Anti-Counterfeiting Applications

**DOI:** 10.3390/nano15201578

**Published:** 2025-10-16

**Authors:** Chuanming Wang, Jigang Wang, Yuansheng Qi, Jindi Hu, Haiming Li, Jianhui Lv, Xiaohan Cheng, Deyu Pan, Zhenjun Li, Junming Li

**Affiliations:** 1 Beijing Key Laboratory of Printing and Packaging Materials and Technology, Beijing Institute of Graphic Communication, Beijing 102600, China; w_chuanming@163.com (C.W.); di13087073292@163.com (J.H.); 17363387595@163.com (H.L.); 17854211937@163.com (J.L.); c1595631742@163.com (X.C.); 18717155359@163.com (D.P.); 2 Laboratory of Nanophotonic Materials and Devices, National Center for Nanoscience and Technology, Beijing 100190, China; 3 Laboratory of Standardization and Measurement for Nanotechnology, National Center for Nanoscience and Technology, Beijing 100190, China; 4 Beijing Key Laboratory for Sensors, Beijing Information Science & Technology University, Beijing 100192, China; li@bistu.edu.cn

**Keywords:** solution combustion synthesis, BaAl_2_O_4_: Eu^2+^, Nd^3+^, Pr^3+^, multi-level authentication, anti-counterfeiting

## Abstract

Solution combustion-synthesized BaAl_2_O_4_: Eu^2+^, Nd^3+^, and Pr^3+^ blue–green long-afterglow phosphors are prepared and systematically investigated. First, XRD confirms the BaAl_2_O_4_ host and screens for trace residual features. SEM reveals the agglomerated granular morphology typical of combustion products. XPS verifies the valence states (Eu^2+^, Nd^3+^, Pr^3+^) and the chemical environment of the host lattice. UV-Vis diffuse reflectance spectra, transformed via the Kubelka–Munk function and analyzed using Tauc plots (indirect-allowed), indicate a wide band gap of the BaAl_2_O_4_ host with small, systematic shifts upon Nd^3+^/Pr^3+^ co-doping. PL measurements show Eu^2+^ 4f–5d emission and co-dopant-assisted excitation/defect pathways without altering the Eu^2+^ emission band shape. Afterglow lifetime and decay analyses correlate trap depth/distribution with the extended persistence. Finally, we demonstrate anti-counterfeiting by (i) snowflake printing and (ii) a binary 3 × 3 grid printed with two afterglow inks of different lifetimes to realize multi-level authentication. The sequential evidence links structure, chemistry, optical absorption, carrier trapping, and practical readout, providing a coherent basis for performance enhancement and application.

## 1. Introduction

Persistent luminescent phosphors (PLPs) store carriers in metastable traps and release them thermally at room temperature, enabling delayed emission for safety signage, bioimaging, information storage, and anti-counterfeiting [1,2,3,4,5]. Among oxide hosts, SrAl_2_O_4_: Eu^2+^, Dy^3+^ is the green benchmark, achieving minute- to hour-scale persistence through trap engineering (rare-earth co-dopants, defect chemistry, atmosphere control) [6,7,8,9]. However, blue-emitting PLPs remain comparatively under-developed, despite advantages for multiplexed/covert security channels, reduced spectral overlap with ambient lighting, and straightforward integration into RGB printing workflows [10,11,12].

BaAl_2_O_4_ is a wide-band-gap aluminate that supports Eu^2+^ 5d–4f emission in the cyan/blue region (~490 nm) and allows trap tailoring via rare-earth co-dopants [13,14,15]. Prior studies have explored Eu^2+^ activation and co-dopants (e.g., Nd^3+^, Pr^3+^), yet gaps persist: (i) limited low-temperature combustion routes that yield fine powders directly compatible with screen-printing inks; and (ii) a shortage of structure–optics–application chains that connect phase/chemistry → optical absorption/band gap (Kubelka–Munk + Tauc) → PL/PLE pathways → afterglow kinetics → device-level readout for authentication [16,17,18]. Moreover, while Sr-aluminate maximizes persistence duration in green emission, it is not necessarily optimal for blue, time-gated, printable security features where rapid “reset” and dual-lifetime encoding are desirable.

This work targets that application space by (i) synthesizing BaAl_2_O_4_: Eu^2+^ and BaAl_2_O_4_: Eu^2+^, Nd^3+^, Pr^3+^ via a 600 °C combustion process, producing ink-ready powders; (ii) establishing a sequential evidence chain—XRD/SEM/XPS → UV-Vis diffuse reflectance (Kubelka–Munk + Tauc) → PL/PLE → afterglow lifetime/decay—to clarify how Nd^3+^/Pr^3+^ modulates trap depths/distributions while preserving the Eu^2+^ emission band; and (iii) demonstrating screen-printed, time-gated anti-counterfeiting with a snowflake motif and a binary 3 × 3 grid using dual-lifetime inks to realize multi-level authentication. We acknowledge that the best persistence here (~34 s to visual threshold) is shorter than optimized SrAl_2_O_4_: Eu^2+^, Dy^3+^ systems (minutes–hours). Our aim is not to surpass Sr-aluminate in absolute duration but to provide a complementary blue channel with low-temperature, printing-compatible processing and time-gated readout suited to dynamic, multilayer security encoding.

## 2. Experiment Content

### 2.1. Materials and Methods

BaAl_2_O_4_: xEu^2+^, yNd^3+^, zPr^3+^ (x = 0, 0.01, 0.02, 0.03, 0.04, 0.05; y = 0.01, 0.02, 0.03, 0.04, 0.05; z = 0.0005, 0.001, 0.0015, 0.0020, 0.0025) series of fluorescent powders. Eu_2_O_3_ (purity: 99.99%), Al_2_O_3_ (purity: 99.99%), BaCO_3_ (purity: 99.99%), Nd_2_O_3_ (purity: 99.99%), Pr(NO_3_)_3_ (purity: 99.99%), HNO_3_ (purity: 80%), and urea (purity: 99.99%) were used as raw materials. All chemicals were sourced from Tianjin Chemical Reagent Factory (Tianjin, China) and employed as received without further drying or purification.

### 2.2. Material Synthesis

First, high-purity Eu_2_O_3_, Al_2_O_3_, BaCO_3_, Nd_2_O_3_, and Pr(NO_3_)_3_ were dissolved in concentrated nitric acid and deionized water. By carefully controlling the molar concentration, the following nitrate solutions were prepared: Ba(NO_3_)_2_ (0.5 mmol/mL), Al(NO_3_)_3_ (1.0 mmol/mL), Eu(NO_3_)_3_ (0.1 mmol/mL), Nd(NO_3_)_3_ (0.5 mmol/mL), and Pr(NO_3_)_3_ (0.5 mmol/mL). The solutions were then combined in stoichiometric proportions corresponding to the target composition BaAl_2_O_4_: xEu^2+^, yNd^3+^, zPr^3+^.

During this process, 2.2 g of urea (CO(NH_2_)_2_) was added as both a reducing agent and combustion promoter. The mixture was magnetically stirred until a homogeneous transparent solution was obtained. The solution was transferred into an alumina crucible and placed in a preheated muffle furnace, where a spontaneous combustion reaction occurred. The reaction was maintained for 5–8 min until completion, producing a loose, porous, foamy white solid. After cooling to room temperature, the product was ground for 20–30 min to obtain a uniform fine powder. The final powders were sealed in test tubes for later use.

Using this solution combustion method, three series of phosphors were synthesized: BaAl_2_O_4_: Eu^2+^, BaAl_2_O_4_: Eu^2+^, Nd^3+^, and BaAl_2_O_4_: Eu^2+^, Nd^3+^, Pr^3+^. The Eu^2+^ concentration was first varied systematically (0–5%) to determine the optimal doping level. Based on this value, Nd^3+^ was introduced in a gradient (0–5%) to investigate its effect. Finally, with the Eu^2+^/Nd^3+^ ratio fixed at the optimized level, Pr^3+^ was added in small amounts (0.05–0.25%) to study its synergistic role.

To further explore the influence of thermal treatment, samples with the optimized dopant concentrations were calcined at temperatures ranging from 500 to 900 °C (in 100 °C intervals). The resulting products were analyzed to evaluate their phase composition, photoluminescence (PL) spectra, and afterglow decay curves. These measurements enabled the determination of the optimal ion doping levels and calcination temperature parameters, and the overall stepwise optimization procedure is summarized in Table 1.

### 2.3. Preparation of Ink

**Figure 1:**
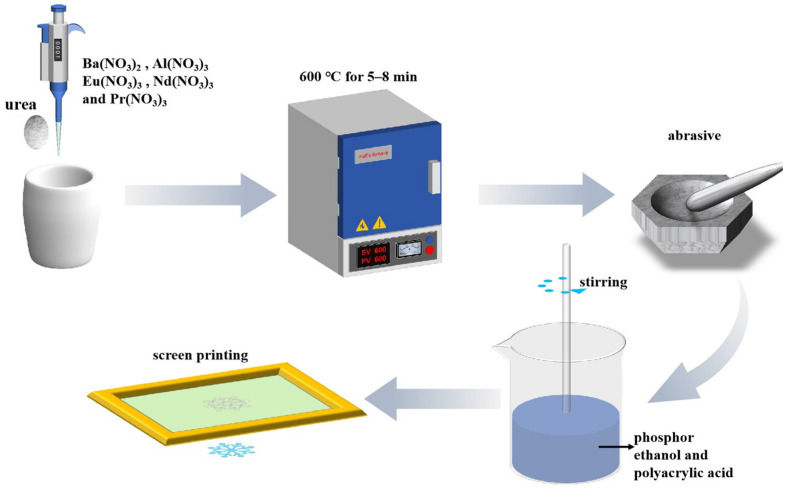
Screen printing ink preparation and transfer process.

The BaAl_2_O_4_: Eu^2+^, Nd^3+^, Pr^3+^ fluorescent powder synthesized by the combustion method was introduced into a mixed solvent system composed of anhydrous ethanol and polyacrylic acid (PAA). The fluorescent powder was colloidally dispersed in the ethanol-PAA system by stirring it with a glass rod, ensuring its rheological properties met the process requirements for screen printing. In this process, PAA acts as a binder, enhancing the ink’s film adhesion and inhibiting fluorescence quenching through the coordination of carboxyl groups with rare-earth ions. The fluorescent ink appears colorless and transparent under visible light but exhibits corresponding colors under UV excitation. The flowchart in Figure 1 systematically analyzes the entire process chain from fluorescent powder synthesis, ink homogenization, and dispersion to screen printing pattern formation.

### 2.4. Characterization of Materials

The crystallographic framework of the as-prepared samples was examined with a Rigaku D/max 2200PC powder X-ray diffractometer (Cu Kα radiation, λ = 1.5406 Å) in the 10–70° (2θ) angular range. The microstructure of the long-afterglow phosphorescent materials was characterized using a field emission scanning electron microscope (Hitachi Quanta 250 FEG, Tokyo, Japan). Elemental composition was analyzed semi-quantitatively using a scanning electron microscope energy dispersive spectrometer (SEM-EDS, Thermo Fisher Scientific, Waltham, MA, USA). Binding energy was determined using an X-ray photoelectron spectrometer (Thermo Scientific ESCALAB 250 XI, Waltham, MA, USA), with the excitation source being monochromatic Al Kα radiation (hν = 1486.6 eV). UV-visible diffuse reflectance spectra were collected using a UV-3600 spectrophotometer (Shimadzu Corporation, Kyoto, Japan). Photoluminescence (PL) spectra and their excitation spectra (PLE) were measured using a fluorescence spectrometer (F4700, Hitachi, Japan). The fluorescence lifetime and afterglow decay curves of long-afterglow phosphors were quantitatively characterized using a transient-steady-state fluorescence spectrometer (Hamamatsu Photonics Quantaurus-Tau C16361-2, Chiyoda, Japan).

## 3. Results and Discussion

### 3.1. XRD Structural Analysis

**Figure 2:**
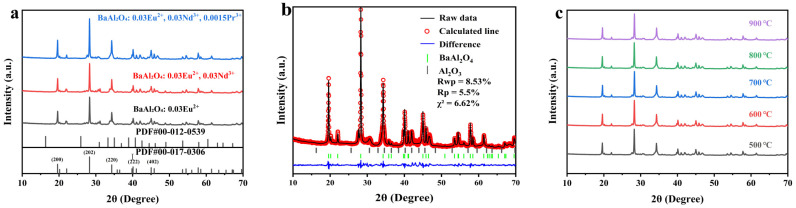
(**a**) X-ray diffraction (XRD) patterns of BaAl_2_O_4_ phosphors doped with different ions; (**b**) Rietveld refinement profile with Bragg markers for BaAl_2_O_4_ and Al_2_O_3_; (**c**) XRD patterns of BaAl_2_O_4_: Eu^2+^, Nd^3+^, Pr^3+^ samples calcined at 500–900 °C.

The effects of rare-earth ion (Eu^2+^, Nd^3+^, Pr^3+^) doping on the crystal structure of BaAl_2_O_4_ fluorescent powders were systematically analyzed using X-ray powder diffraction (XRD, Cu-Kα radiation, λ = 1.5406 Å). As shown in Figure 2a, all samples exhibit significant diffraction peaks at 2θ = 19.602° (d = 4.53 Å), 28.282° (d = 3.15 Å), 34.317° (d = 2.61 Å), 40.115° (d = 2.25 Å), and 45.042° (d = 2.01 Å). These peak positions are consistent with the characteristic diffraction peaks of the hexagonal phase BaAl_2_O_4_ standard card (PDF#00-017-0306, space group P6_3_22) [19]. Figure 2a indicates that the diffraction peak positions exhibit no observable shift, indicating that the doping of Eu^2+^, Nd^3+^, and Pr^3+^ ions has not altered the crystalline phase of the BaAl_2_O_4_ matrix.

We conducted Rietveld refinement on the XRD patterns for BaAl_2_O_4_: Eu^2+^, Nd^3+^, Pr^3+^ long-afterglow phosphors synthesized via calcination at 600 °C employing the General Structure Analysis System-II (GSAS-II, v5.3.3, Revision #5806) software. Presented in Figure 2b, the experimental data exhibit high consistency with the fitted curve, with no major unidentified reflections. However, a small number of faint residual reflections can still be identified, indicating the presence of trace impurity phases. For the BaAl_2_O_4_: Eu^2+^, Nd^3+^, Pr^3+^ sample, the Rietveld refinement results show that the measured Rwp value is 8.53%. The Rp value is 5.5%, which is less than 10%, indicating that the sample has good physical and mathematical agreement with the standard card data, further confirming the hexagonal phase structure (PDF#00-017-0306) and purity of the sample. Eu^2+^ and Nd^3+^ ions tend to occupy the Ba^2+^ positions in the BaAl_2_O_4_ main lattice [20,21], thereby introducing a doping effect. However, the doping concentration of Pr^3+^ is low (0.15%), and it may also exert a particular influence on the lattice structure. In the full-range pattern (Figure 2a), an impurity peak is observed at 2θ = 31.0°. Based on JADE analysis, Figure 2a overlays Al_2_O_3_ (PDF #00-012-0539); three residual reflections—including the ~31° line—match the Al_2_O_3_ sticks within ± 0.1–0.2° (2θ), and the feature is therefore assigned to a trace Al_2_O_3_ secondary phase. Quantitative Rietveld analysis in GSAS-II yields an Al_2_O_3_ fraction of ~1.3 wt.%, confirming that the impurity level is minor. For clarity, Figure 2b has been updated to show Bragg reflection markers for Al_2_O_3_. This Al_2_O_3_ most likely results from incomplete reaction inherent to the combustion-synthesis process. Figure 2c shows the XRD patterns of BaAl_2_O_4_: 0.03Eu^2+^, 0.03Nd^3+^, 0.0015Pr^3+^ nanophosphors obtained by calcination at different temperatures. The diffraction peaks’ positions are essentially unchanged in comparison to Figure 2a, suggesting that the crystal phase of the BaAl_2_O_4_ host is not affected by the co-doping of Eu^2+^, Nd^3+^, Pr^3+^ ions.

Based on the XRD spectrum data, we used Origin 2024 software to perform Gaussian fitting on the diffraction peaks to extract key parameters such as peak position (expressed in radians) and full width at half maximum (FWHM). The grain size was calculated using the Scherrer formula [22]:

(1) $$D=\frac{k\lambda }{\beta \cos\theta }$$

Among these, k is the Scherrer constant (set to 0.89), λ is the X-ray wavelength (0.15406 nm), and β and θ correspond to the full width at half maximum (FWHM) (in radians) and the diffraction angle of the corresponding peak, respectively. All calculations were performed using Origin software. We further determined the sample’s lattice spacing, microstrain, and dislocation density, with corresponding results tabulated in Table 2.

### 3.2. SEM Analysis

**Figure 3:**
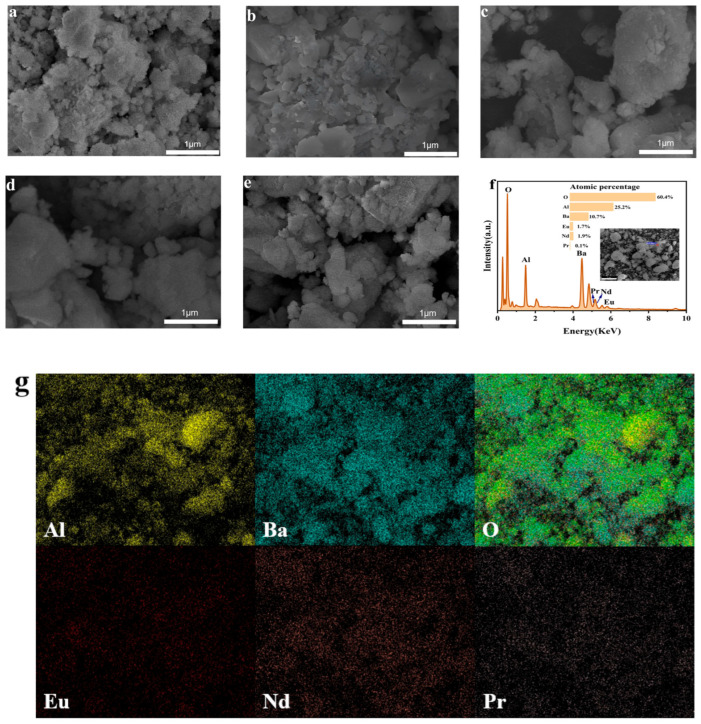
SEM images of BaAl_2_O_4_: Eu^2+^, Nd^3+^, Pr^3+^ synthesized at different temperatures: (**a**) 500 °C, (**b**) 600 °C, (**c**) 700 °C, (**d**) 800 °C, (**e**) 900 °C; (**f**) EDS analysis results of BaAl_2_O_4_: 0.03Eu^2+^, 0.03Nd^3+^, 0.0015Pr^3+^ powder prepared at 600 °C; (**g**) SEM–EDS elemental maps of the BaAl_2_O_4_: Eu^2+^, Nd^3+^, Pr^3+^ sample showing the spatial distributions of Al, Ba, O (top row) and rare-earth dopants Eu, Nd, and Pr (bottom row).

SEM images (Figure 3a–e) reveal temperature-dependent morphology of BaAl_2_O_4_: Eu^2+^, Nd^3+^, Pr^3+^. At 500–600 °C, irregular nanoparticles form a mesoporous network. With rising temperature (700–800 °C), particles coalesce and locally densify, and by 900 °C submicron agglomerates dominate with reduced porosity. This morphological evolution is attributed to vigorous gas release (NO_x_, CO_2_) during combustion, which generates transient escape channels and leaves residual pores [23]. The particle size exhibits non-uniformity, which is attributed to the thermal gradient effect at the combustion front leading to localized nucleation, imbalance in solute diffusion caused by changes in the precursor solution [24], and uneven distribution of mechanical stress during grinding [25]. At elevated temperature, the hexagonal BaAl_2_O_4_ lattice (P6_3_22) promotes bulk agglomeration, while the remaining pores can act as diffusion pathways for rare-earth ions, affecting their lattice incorporation efficiency [26,27,28].

The detection of Ba, Al, O, Eu, Nd, and Pr has been confirmed through EDS analysis of the BaAl_2_O_4_: 0.03Eu^2+^, 0.03Nd^3+^, 0.0015Pr^3+^ sample (Figure 3f). Local micro-area analysis further revealed the elemental composition of the sample and its corresponding characteristic X-ray intensity signals [29]. Although the chemical composition of the sample generally aligns with the EDS spectral intensity, there is a certain deviation between the actual doping ratios of Eu, Nd, and Pr and the expected stoichiometric ratios [30]. This deviation may stem from the solution combustion synthesis method employed, which completes the doping process through rapid redox reactions of the precursor solution in an extremely short time, leading to intense exothermic reactions. This could lead to non-uniform elemental distribution within the crystal lattice, causing segregation phenomena.

Due to the low doping concentration of Pr^3+^ (only 0.15%), its characteristic peak intensity in the EDS spectrum (Figure 3f) is relatively weak, with significant overlap with the Nd^3+^ peak. The intensity difference between the two is slight, which, to some extent, increases the difficulty of quantitative analysis of low-concentration elements. Nevertheless, EDS can still clearly detect the characteristic peaks of Eu^2+^, Nd^3+^, and Pr^3+^, indicating that all three have successfully entered the crystal lattice structure of BaAl_2_O_4_. As an orthogonal check, SEM–EDS elemental maps (Figure 3g) show a reproducible Pr distribution above background, co-localized with the Ba/Al/O host and without RE-rich segregation at the micron scale. Eu and Nd maps exhibit similar uniformity. Given the Nd/Pr L-line overlap, conventional EDS is unsuitable for quantitative Pr/Nd partitioning. We therefore use EDS only for above-background presence and colocalization, while XPS provides chemically specific identification and oxidation states.

### 3.3. XPS Analysis

**Figure 4:**
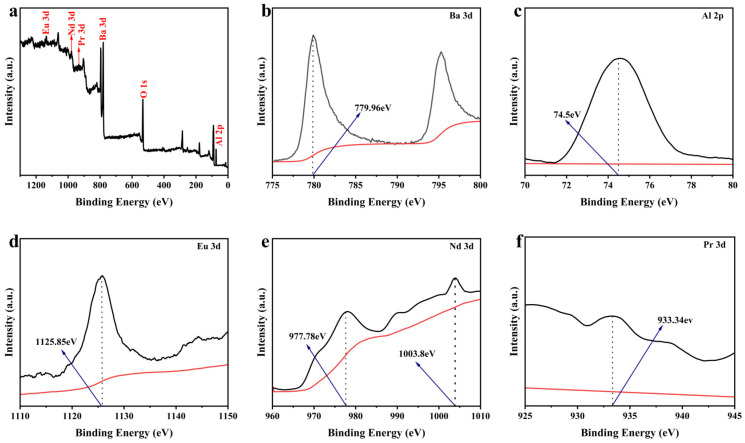
(**a**) Full XPS spectrum of BaAl_2_O_4_: Eu^2+^, Nd^3+^, Pr^3+^; (**b**–**f**) High-resolution XPS spectra of Ba 3d, Al 2p, Eu 3d, Nd 3d, and Pr 3d (black lines represent the experimental XPS data, and red lines correspond to the fitted curves used for peak deconvolution).

In this study, to systematically evaluate the chemical valence states of Eu^2+^, Nd^3+^, and Pr^3+^ ions in a BaAl_2_O_4_ matrix and their effects on the material’s luminescent properties, we characterized of samples using X-ray photoelectron spectroscopy (XPS) technology. XPS is a surface-sensitive analytical method that provides information on elements’ chemical composition, oxidation state, and chemical environment [31]. Through full-spectrum scanning and high-resolution narrow-band scanning, we successfully identified the characteristic binding energy peaks of the main elements (Ba, Al, O, Eu, Nd, Pr) in the samples, as shown in Figure 4.

As shown in Figure 4a, XPS measurement results reveal binding energy peaks corresponding to Ba 3d, Al 2p, O 1s, Eu 3d, Nd 3d, and Pr 3d, indicating that these elements are present in the sample [32,33]. Further high-resolution scanning analysis revealed that the binding energy peak of Eu 3d was 1125.85 eV, consistent with the characteristic binding energy of Eu^2+^ (3d_5/2_), indicating that Eu^2+^ exists in the sample in its divalent form [34,35,36]. Additionally, the binding energy peak of Nd 3d is located at 977.78 eV and 1003.8 eV, consistent with the typical binding energy range of Nd^3+^, indicating that Nd^3+^ also maintains its expected trivalent state [37,38,39]. Low-concentration Pr^3+^ doping (0.15%) results in a weak characteristic peak, with a binding energy peak at 933.34 eV. High-resolution scanning confirms the presence of the Pr 3d characteristic peak, demonstrating that Pr^3+^ has been doped into the BaAl_2_O_4_ lattice and maintains its trivalent state [40,41]. XPS confirmed that all doped ions are in the target valence state, a result that directly supports subsequent studies on the luminescent properties of the material.

### 3.4. Ultraviolet Diffuse Reflectance Characterization

**Figure 5:**
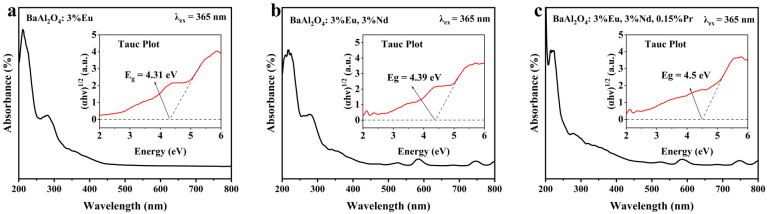
(**a**–**c**) UV-Vis absorption spectra of (**a**) BaAl_2_O_4_: Eu^2+^; (**b**) BaAl_2_O_4_: Eu^2+^, Nd^3+^, and (**c**) BaAl_2_O_4_: Eu^2+^, Nd^3+^, Pr^3+^. Insets: Tauc plots for indirect-allowed transitions.

UV-Vis diffuse reflectance spectroscopy (UV-Vis) was employed to characterize the long-afterglow phosphors BaAl_2_O_4_: Eu^2+^, BaAl_2_O_4_: Eu^2+^, Nd^3+^, and BaAl_2_O_4_: Eu^2+^, Nd^3+^, Pr^3+^ synthesized at 600 °C, with a measurement range of 200–800 nm, to determine their optical bandgap values. Figure 5a–c show the UV-Vis diffuse reflectance spectra of the three samples, respectively. According to the Tauc formula [42,43]:

(2) $$(\alpha \mathrm{hv}{)}^{1/2}\;=\;A(h\nu \;-\;E_{g})$$

Among these, A is a material-related constant, E_g_ is the bandgap value, hν is the photon energy, and α is the light absorption coefficient. By plotting (αhν)^1/2^ against hv and extrapolating to (αhν)^1/2^ = 0, the bandgap values for BaAl_2_O_4_: Eu^2+^, BaAl_2_O_4_: Eu^2+^, Nd^3+^, and BaAl_2_O_4_: Eu^2+^, Nd^3+^, Pr^3+^ are determined to be 4.31 eV, 4.39 eV, and 4.5 eV, respectively. The incorporation of Eu^2+^, Nd^3+^, and Pr^3+^ does not significantly alter the host band gap, indicating that the electronic structure of BaAl_2_O_4_ is largely preserved. We note that these numbers represent the Tauc onset rather than a rigid shift of the host band edges. The modest widening upon Nd^3+^ and Nd^3+^/Pr^3+^ co-doping is attributed to a reduction in sub-gap/tail-state absorption and edge sharpening due to defect re-distribution and charge-compensation by trivalent co-dopants, together with fewer non-radiative centers; effectively, the co-dopants clean the absorption edge, yielding a slightly larger apparent Tauc gap while leaving the Eu^2+^ emission band shape essentially unchanged. This stability is beneficial for sustaining its long afterglow luminescence performance.

Upon Nd^3+^/Pr^3+^ co-doping, the absorbance in the 270–280 nm segment of the Eu^2+^ 4f → 5d envelope decreases. This behavior is attributed to the suppression of sub-gap/Urbach-tail and defect-related absorption by charge-compensated defect complexes, together with a slight redistribution of oscillator strength due to local crystal-field modulation at Eu^2+^ sites. The cleaner near-edge response is consistent with the modest blue shift of the apparent edge (4.31 → 4.39 → 4.5 eV), while the wide band gap of the host remains essentially preserved.

Diffuse reflectance R(λ) was converted to the Kubelka–Munk function F(R) = (1−R)^2^/(2R). To quantitatively characterize the edge steepness within this section, we further employed the Urbach-tail formalism on the near-edge exponential region: linear fits of lnA (absorbance used as a surrogate of α) versus photon energy hν were performed below the respective Tauc onsets to avoid interference with the Tauc linear segment. The Urbach energy was extracted as E_U_ = 1/slope. For each composition we report the fit window (eV), data-point count, least-squares R^2^, and 95% confidence interval (CI) of E_U_; the dimensionless steepness index S = kT/E_U_ (300 K) is also listed. The corresponding data is shown in Table 3.

All Urbach fits were carried out below the corresponding Tauc onsets (4.31/4.39/4.50 eV); therefore, these model-based metrics quantify edge steepness without altering or contradicting the bandgap values reported above. Within this framework, Nd co-doping yields the steepest/cleanest edge (smallest E_U_), while additional Pr slightly increases the tail (E_U_ rises).

### 3.5. Photoluminescence Analysis

**Figure 6:**
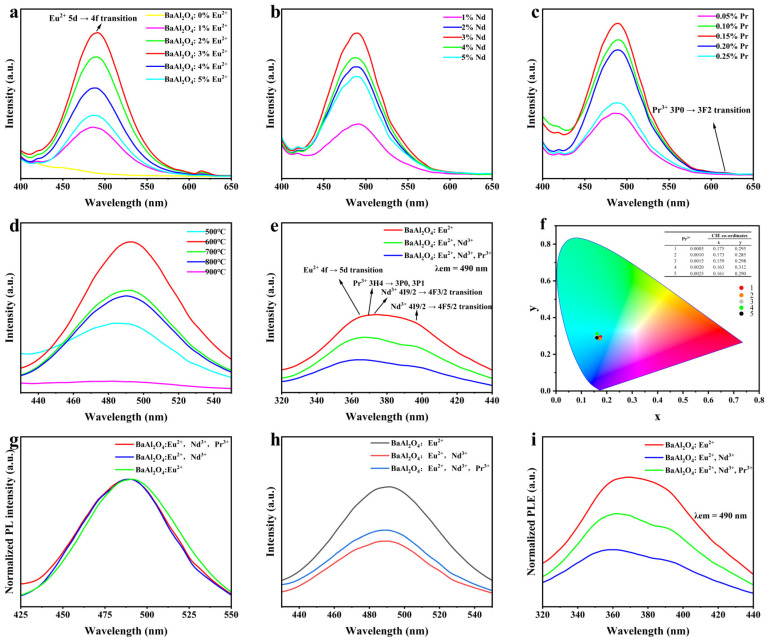
PL/PLE of BaA_2_O_4_-based phosphors: (**a**) Eu^2+^ concentration (λex = 365 nm); (**b**) Nd^3+^ co-doping; (**c**) Pr^3+^ co-doping; (**d**) calcination temperature (500–900 °C); (**e**) PLE; (**f**) CIE map; (**g**,**h**) normalized/raw PL; (**i**) normalized PLE.

Figure 6 shows the influence of ion concentration and temperature upon the luminescence characteristics of BaAl_2_O_4_ phosphors. Figure 6a displays the emission spectrum of BaAl_2_O_4_: xEu^2+^ (x = 0–0.05) samples sintered at 600 °C under 365 nm excitation, exhibiting a broad blue-green emission peak at 490 nm, originating from the Eu^2+^ 4f^6^5d^1^ → 4f^7^ transition [44,45,46,47]. As the Eu^2+^ concentration increases, the luminescence intensity enhances, reaching a peak at x = 0.03 before undergoing concentration quenching. The emission peak position (490 nm) remains unchanged across different x values, indicating a stable lattice field environment.

Figure 6b investigates the effect of Nd^3+^ co-doping (Ba_0.97-y_Al_2_O_4_: 0.03Eu^2+^, yNd^3+^, y = 0–0.05). When y = 0.03, the emission intensity at 490 nm is maximum. Nd^3+^ ions introduce trap centers in the BaAl_2_O_4_ matrix. These centers enhance the afterglow by facilitating energy transfer to Eu^2+^ and serve as reservoirs for controlled carrier release [48,49,50].

Figure 6c shows the effect of Pr^3+^ doping (Ba_0.94-z_Al_2_O_4_: 0.03Eu^2+^, 0.03Nd^3+^, zPr^3+^, z = 0.0005–0.0025). The best luminescence effect is observed at z = 0.0015, where Pr^3+^ introduces additional traps and facilitates more efficient energy transfer between Eu^2+^ and Nd^3+^. This synergistic role enhances the afterglow and suppresses non-radiative losses [49,51], thereby improving the overall PL performance [49,50]. Deviating from this concentration leads to insufficient defects or concentration quenching [48,50].

Based on the optimized dopant concentrations (Eu^2+^: 0.03; Nd^3+^: 0.03; Pr^3+^: 0.0015), Figure 6d depicts the effect of sintering temperature (500–900 °C) on the luminescence performance. The sample sintered at 600 °C exhibits the highest emission intensity at 490 nm. However, the luminescence intensity progressively decreases when the temperature exceeds 800 °C. This reduction is likely due to increased lattice distortion at elevated temperatures, destabilizing the luminescent centers and reducing energy transfer efficiency.

Figure 6e presents the photoluminescence excitation (PLE) spectrum of the sample, showing a broad excitation band centered around 365 nm, corresponding to the 4f^7^ → 4f^6^5d^1^ transition of Eu^2+^ ions. This indicates that 365 nm ultraviolet light is an effective excitation source, consistent with the emission spectra in Figure 6a–d.

The CIE 1964 Supplementary Standard Colorimetric System (10° field of view) defines a complete geometric model for color quantification in visible light colorimetry. The associated chromaticity diagram exhibits a horseshoe-shaped spectral locus, comprising chromaticity coordinates of monochromatic light (380–780 nm) with boundary points representing saturated spectral colors. The central white point (x_10_ = 0.3333, y_10_ = 0.3333) corresponds to equi-energy white light. Color saturation progressively diminishes toward this central point. At the same time, hue differences are quantified azimuthally—radial lines extending from the white point intersect the spectral locus at coordinates corresponding to dominant wavelengths. Chromaticity coordinates (x_10_, y_10_) are derived from normalized tristimulus values (X_10_, Y_10_, Z_10_) as follows [52]:

(3) $$x=\frac{X}{X+Y+Z}$$

(4) $$y=\frac{Y}{X+Y+Z}$$

Among these, X, Y, and Z are the CIE three-stimulus values, obtained by integrating the color stimulus function with the color matching function of the CIE 1964 color space system. This model converts human color perception into quantitative parameters, improving accuracy as the field of view increases. It is the core standard for material luminescence analysis and industrial color difference detection. This study calculated spectral data for samples with different Pr^3+^ doping concentrations, yielding corresponding CIE 1964 xy chromaticity coordinates (Figure 6f). The chromaticity coordinates of all five concentration samples are concentrated in the blue-green light region, indicating that Pr^3+^ doping has not altered the material’s emission color, consistent with the 490 nm emission peak position.

To clarify the excitation pathways and the effects of co-doping, we uniformly processed the PL/PLE data for the three compositions. Figure 6i presents the normalized PLE spectra monitored at the Eu^2+^ emission (490 ± 10 nm). After normalization to the maximum, the overall response follows Eu > Eu + Nd > Eu + Nd + Pr, indicating that Nd^3+^/Pr^3+^-related traps compete for excited carriers and thereby divert part of the excitation from being immediately fed to Eu^2+^ under steady-state monitoring. In contrast, the normalized PL spectra (λex = 365 nm) in Figure 6g nearly overlap in λ_max and FWHM, demonstrating that the Eu^2+^ emission band is preserved and that co-doping mainly affects excitation/trapping and non-radiative channels, rather than altering the local crystal field at the Eu^2+^ site. For independent verification, the raw (non-normalized) PLE and PL spectra corresponding to Figure 6e,h are shown here for absolute-intensity comparison.

The luminescence mechanism of BaAl_2_O_4_-based phosphors is similar to that of Sr/CaAl_2_O_4_ systems, especially in the presence of Eu^2+^ and rare-earth ions. The luminescence of Eu^2+^ originates from the transition from the ground state 4f^6^ to the excited state 5d^1^. When Nd^3+^, Pr^3+^, and other ions are incorporated, these ions provide deep-level traps in the system, which delay electron recombination and significantly extend the afterglow time [53,54,55,56].

In the BaAl_2_O_4_: Eu^2+^, Nd^3+^, Pr^3+^ system, Nd^3+^ and Pr^3+^ not only provide luminescent centers but also create additional electron trapping sites. These traps slow down the electron recombination process, thereby enhancing the afterglow properties. Furthermore, the energy transfer mechanism between Eu^2+^ and Nd^3+^ explains how these materials can achieve prolonged afterglow luminescence [57,58].

The luminescence mechanism of BaAl_2_O_4_-based phosphors primarily relies on the 5d–4f transition of Eu^2+^ and its interaction with deep-level traps. The incorporation of Nd^3+^ and Pr^3+^ ions further improves the efficiency of electron trapping and release, thus achieving long-lasting afterglow luminescence.

### 3.6. Fluorescence Lifetime and Afterglow Decay

**Figure 7:**
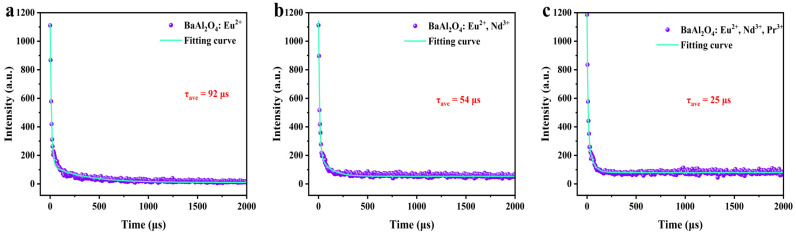
(**a**–**c**) shows the fluorescence lifetime decay curves and fitting curves of BaAl_2_O_4_: Eu^2+^, BaAl_2_O_4_: Eu^2+^,Nd^3+^, and BaAl_2_O_4_: Eu^2+^, Nd^3+^, Pr^3+^ phosphors.

The fluorescence decay of BaAl_2_O_4_: Eu^2+^, BaAl_2_O_4_: Eu^2+^, Nd^3+^, and BaAl_2_O_4_: Eu^2+^, Nd^3+^, Pr^3+^ samples under UV excitation was tested. Figure 7 shows these samples’ decay curves and fitting results under 365 nm excitation. All samples exhibited double-exponential decay, consisting of fast and slow components. The double-exponential function fitting using Equation (5) was employed, where I_0_ is the background constant, A_1_ and A_2_ are constants, t is the decay time, and τ_1_ and τ_2_ are the decay times of the exponential components [49]. These parameters were calculated using Origin software, with specific values in Table 4. The average decay time τ * of the samples can be calculated using Equation (6) [59].

(5) $$I(t)=I_{0}+A_{1}\exp(-t/\tau_{1})+A_{2}\exp(-t/\tau_{2})$$

(6) $$\tau_{\mathrm{ave}}=\frac{A_{1}\tau_{1}^{2}+A_{2}\tau_{2}^{2}}{A_{1}\tau_{1}+A_{2}\tau_{2}}$$

The fast component is primarily attributed to the intrinsic radiative relaxation of the Eu^2+^ centers (5d → 4f transition) and the rapid detrapping of carriers from shallow traps. In contrast, the slow component reflects population stored in deeper traps, which are released slowly either by thermally activated detrapping or by tunneling through the traps before recombination at Eu^2+^ centers. This dual decay process—rapid release from shallow traps and slower release from deep traps—is consistent with the known models for Eu^2+^-activated aluminates.

The average decay times calculated using the above formula are as follows: BaAl_2_O_4_: Eu^2+^ is 92 μs, BaAl_2_O_4_: Eu^2+^, Nd^3+^ is 54 μs, and BaAl_2_O_4_: Eu^2+^, Nd^3+^, Pr^3+^ is 25 μs. It can be observed that the introduction of dopant ions significantly reduces the fluorescence lifetime of the samples. This may be attributed to Nd^3+^ replacing some Ba^2+^ sites in the matrix, forming trap centers with suitable energy levels that effectively capture some excited-state electrons. Pr^3+^ further enhances the trap effect, increasing electron capture efficiency and exacerbating concentration quenching. The dopant ions effectively regulate the fluorescence lifetime of the material.

**Figure 8:**
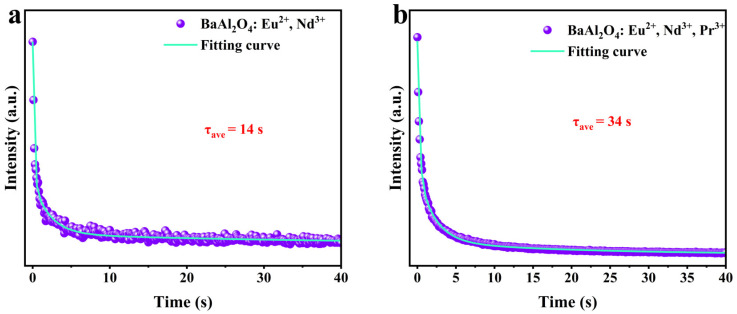
Afterglow decay curves and fitting results of (**a**) BaAl_2_O_4_: Eu^2+^, Nd^3+^ and (**b**) BaAl_2_O_4_: Eu^2+^, Nd^3+^, Pr^3+^ after 3 min of 365 nm UV excitation.

The afterglow decay of BaAl_2_O_4_: Eu^2+^, Nd^3+^ and BaAl_2_O_4_: Eu^2+^, Nd^3+^, Pr^3+^ samples was measured under 365 nm excitation. The experimental data were fitted using a double exponential function, with the results shown in Table 5. The afterglow decay consists of two stages: a fast decay dominates the initial intensity decline, while the slow decay corresponds to extended afterglow emission [60]. As shown in Figure 8, the average afterglow time τ for BaAl_2_O_4_: Eu^2+^, Nd^3+^ is 14 s, while that for BaAl_2_O_4_: Eu^2+^, Nd^3+^, Pr^3+^ is extended to 34 s.

Pr^3+^ doping enhances trap density and optimizes the electron capture and release process, improving afterglow performance. The deeper energy level traps formed extend the electron storage time, significantly prolonging the afterglow lifetime. Pr^3+^ doping plays a critical role in modifying the trap properties by introducing deeper energy level traps. These traps effectively capture more electrons, thereby prolonging the afterglow. Additionally, the increased trap density in the system, due to Pr^3+^ codoping, enhances the overall electron capture and release efficiency, contributing to the extended afterglow times observed in BaAl_2_O_4_: Eu^2+^, Nd^3+^, Pr^3+^ samples. Anti-counterfeiting labels based on such phosphors enhance reliability and ease of identification and also have application potential in the fields of safety emergency indication and optical information storage [61].

The afterglow times observed in BaAl_2_O_4_: Eu^2+^, Nd^3+^ (14 s) and BaAl_2_O_4_: Eu^2+^, Nd^3+^, Pr^3+^ (34 s) are relatively short compared to the long afterglow times seen in SrAl_2_O_4_-based phosphors (such as SrAl_2_O_4_:Eu^2+^, Dy^3+^), where afterglow persists for tens of minutes. The shorter afterglow times observed in BaAl_2_O_4_-based phosphors highlight the influence of the host matrix and the trap distribution on the afterglow performance. However, by optimizing trap properties through codoping with Nd^3+^ and Pr^3+^, the afterglow duration in BaAl_2_O_4_-based phosphors can be significantly improved.

The Eu^2+^ 5d → 4f fluorescence lifetime decreases upon Nd^3+^ and Pr^3+^ co-doping (92 → 54 → 25 µs), whereas the afterglow time increases (14 → 34 s). This behavior is consistent with a branching-kinetics picture. Co-dopants introduce efficient trap-capture channels that compete with prompt emission on the microsecond timescale; hence the observed lifetime follows

(7) $$\tau_{\mathrm{obs}}^{-1}=\tau_{r}^{-1}+\tau_{nr}^{-1}+k_{ET\to traps}$$

and shortens as *k*_ET→*traps*_ increases. In contrast, persistent luminescence is governed by the release of carriers from traps on much longer timescales,

(8) $$I_{afterglow}(t)\propto \sum_{i}n_{i}(0)exp(-t/\tau_{t,i})$$

A back-of-the-envelope estimate using the measured lifetimes yields incremental fast-capture rates of Δk ≈ 7.6 × 10^3^ s^−1^ for Eu → Eu + Nd (92 → 54 µs) and Δk ≈ 2.9 × 10^4^ s^−1^ for Eu → Eu + Nd + Pr (92 → 25 µs), evidencing enhanced diversion of carriers into traps. While this quenches the prompt Eu^2+^ channel, it increases the initial trapped population n_i_(0) and, with Pr^3+^, optimizes the distribution of thermally addressable traps at 300 K, thereby extending the macroscopic afterglow. Prompt PL lifetime and persistent luminescence thus probe different segments of the relaxation network and need not exhibit parallel trends.

To provide a clearer comparison with the existing materials, we have compared the fluorescence lifetimes and afterglow decay times of our BaAl_2_O_4_: Eu^2+^, Nd^3+^, Pr^3+^ phosphor with SrAl_2_O_4_: Eu, Nd system, as shown in Table 6.

As shown in the table, our BaAl_2_O_4_: Eu^2+^, Nd^3+^, Pr^3+^ samples exhibit fluorescence lifetimes of 92 μs, 54 μs, and 25 μs, and afterglow decay times of 0 s, 14 s, and 34 s for Eu^2+^, Eu^2+^, Nd^3+^, and Eu^2+^, Nd^3+^, Pr^3+^, respectively. In comparison, SrAl_2_O_4_: Eu, Nd has fluorescence lifetimes of 404 ns and 46 ns, with afterglow decay times of 0 s and 13 s, respectively [62].

While the BaAl_2_O_4_: Eu^2+^, Nd^3+^, Pr^3+^ system demonstrates shorter afterglow times compared to SrAl_2_O_4_-based materials, the afterglow times in our system still show promising results, demonstrating the effectiveness of the trap-assisted luminescence mechanism. Moreover, our system offers the advantage of lower synthesis temperatures and more controlled emission wavelengths, making it more versatile for specific applications, including anti-counterfeiting and security labeling.

To probe the role of traps in the afterglow, we performed 2D thermoluminescence (2D-TL) on BaAl_2_O_4_: Eu^2+^, BaAl_2_O_4_: Eu^2+^, Nd^3+^, and BaAl_2_O_4_: Eu^2+^, Nd^3+^, Pr^3+^ (pre-irradiation 365 nm; 300–600 K; heating rate β = 3 K s^−1^; detection 300–580 nm, spectrally integrated to 1D glow curves). The TL envelopes change markedly upon co-doping. We quantified the dominant peak of each curve using the high-temperature half-width (δ) method (single-rate, first-order effective estimate), obtaining trap depths of 0.296 eV for BaAl_2_O_4_: Eu^2+^, 0.106 eV for BaAl_2_O_4_: Eu^2+^, Nd^3+^, and 0.109 eV for BaAl_2_O_4_: Eu^2+^, Nd^3+^, Pr^3+^. In the same 300–600 K window, the high-T half-widths increase from 35.25 K (Eu^2+^) to 73.14 K (Eu^2+^, Nd^3+^) and 71.07 K (Eu^2+^, Nd^3+^, Pr^3+^), while the integrated TL areas rise from 5.07 × 10^3^ (Eu^2+^) to 2.90 × 10^4^ (Eu^2+^, Nd^3+^) and 1.06 × 10^5^ a.u.·K (Eu^2+^, Nd^3+^, Pr^3+^). The dominant-peak temperatures are 348 K (Eu^2+^) and 300 K (Nd/Pr-containing), with local maxima in the 314–341 K range [63,64].

Taken together, these broad-feature descriptors (broader half-widths and much larger areas) and the δ-based trap depths are consistent with Nd/Pr co-doping producing a broader and more effective trap manifold, with only modest changes in the dominant-peak depth but a substantial increase in trap population/retrapping effectiveness. This picture accounts for the observed trend in persistence (Eu^2+^ < Eu^2+^, Nd^3+^ < Eu^2+^, Nd^3+^, Pr^3+^) via trap-assisted luminescence, without overstating what can be inferred from the present single-rate, 300–600 K dataset.

**Figure 9:**
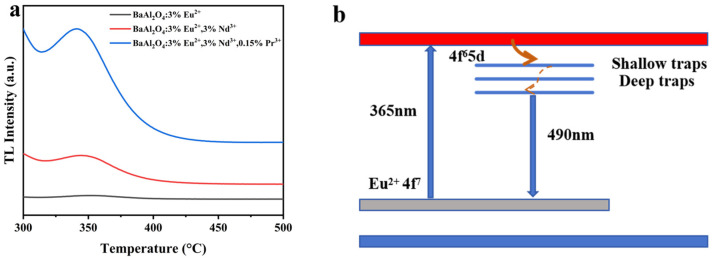
(**a**) Thermoluminescence spectra of BaAl_2_O_4_: Eu^2+^ (black), Eu^2+^, Nd^3+^ (red), and Eu^2+^, Nd^3+^, Pr^3+^ (blue). (**b**) Energy-level diagram showing 365 nm excitation, Eu^2+^ emission (~490 nm), and trap-assisted afterglow via Nd^3+^ shallow and Pr^3+^ deep traps.

As illustrated in Figure 9b, under 365 nm excitation electrons are promoted to the conduction band (CB) and partially relax non-radiatively to the Eu^2+^ 4f^6^5d level, from which radiative recombination to the Eu^2+^ 4f^7^ ground state yields the ≈490 nm emission. In the co-doped samples, Nd^3+^ creates shallower traps whereas Pr^3+^ introduces deeper traps below the CB. Electrons captured by these traps are thermally released and repopulate the Eu^2+^ 5d state, sustaining the persistent luminescence. Consistent with this picture, the average afterglow time increases from ≈14 s (Eu^2+^, Nd^3+^) to ≈34 s (Eu^2+^, Nd^3+^, Pr^3+^), while the BaAl_2_O_4_ host bandgap remains ≈4.3–4.5 eV.

### 3.7. Anti-Counterfeiting Applications

**Figure 10:**
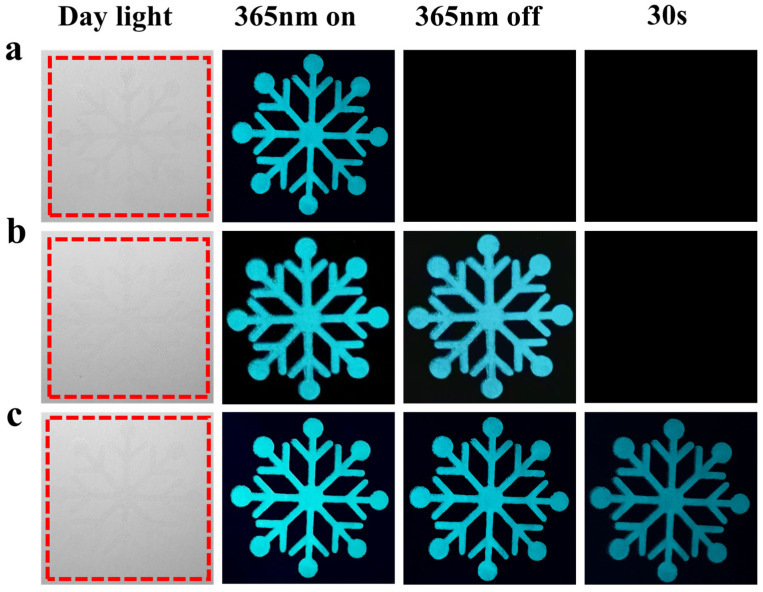
The (**a**) snowflake patterns printed with BaAl_2_O_4_: Eu^2+^ ink; the (**b**) snowflake patterns printed with BaAl_2_O_4_: Eu^2+^, Nd^3+^ ink; the (**c**) snowflake patterns printed with BaAl_2_O_4_: Eu^2+^, Nd^3+^, Pr^3+^.

BaAl_2_O_4_: Eu^2+^, BaAl_2_O_4_: Eu^2+^, Nd^3+^, and BaAl_2_O_4_: Eu^2+^, Nd^3+^, Pr^3+^ phosphors were mixed with polyacrylic acid and ethanol to prepare screen-printed anti-counterfeiting ink, which was used to print a snowflake pattern (Figure 10). Under daylight, the pattern shows no obvious fluorescence, but it exhibits clear luminescence under 365 nm UV light. Upon removal of the UV light source, the fluorescence of BaAl_2_O_4_: Eu^2+^ disappears rapidly, while the BaAl_2_O_4_: Eu^2+^, Nd^3+^ and BaAl_2_O_4_: Eu^2+^, Nd^3+^, Pr^3+^ samples exhibit persistent afterglow. The doping of Nd^3+^ and Pr^3+^ significantly enhances the material’s luminescent properties, increasing its application value in anti-counterfeiting printing.

**Figure 11:**
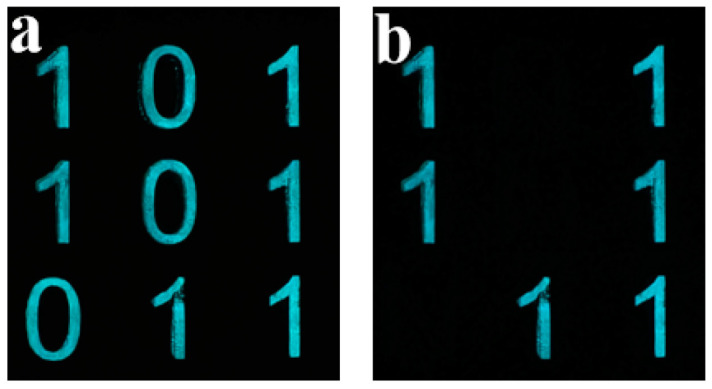
Time-gated dual-lifetime binary grid. (**a**) UV ON/0–2 s after OFF: Code = 365. (**b**) ~14 s after OFF: only long-lifetime cells remain, Code = 63.

We further demonstrate a 3 × 3 lifetime-encoded grid using two inks (≈14 s vs. ≈34 s) (Figure 11). Readout is equation-free: inspect cells in fixed order (weights 1–256) to obtain a decimal code at two time windows (e.g., 365 → 63). Camera exposure and binarization thresholds were kept constant.

### 3.8. Comparative Landscape and Application Positioning

Table 7 summarizes key features of BaAl_2_O_4_, SrAl_2_O_4_, and CaAl_2_O_4_ persistent phosphors relevant to printing and anti-counterfeiting. In brief, SrAl_2_O_4_:Eu^2+^, Dy^3+^ remains the green, long-duration benchmark under optimized high-temperature solid-state synthesis, whereas Ca-aluminates offer blue emission with routes ranging from solid-state to combustion. Our BaAl_2_O_4_: Eu^2+^, Nd^3+^, Pr^3+^ targets a blue/cyan channel produced at 600 °C by combustion, yielding ink-compatible powders and a time-gated, dual-lifetime readout (365 → 63 code pair) suited for multilayer authentication. Rather than maximizing absolute duration, the present work emphasizes low thermal budget, printing compatibility, and lifetime-encoded authentication [65,66,67,68].

## 4. Conclusions

This work elucidates how Nd^3+^/Pr^3+^ co-doping reshapes trap distributions in BaAl_2_O_4_: Eu^2+^ without shifting the Eu^2+^ 4f–5d emission. Indirect-allowed (*n* = 1/2) Tauc plots give band gaps of 4.31, 4.39, and 4.50 eV for Eu^2+^, Eu^2+^/Nd^3+^, and Eu^2+^/Nd^3+^/Pr^3+^, respectively. Prompt PL lifetimes shorten (92 → 54 → 25 μs), whereas macroscopic afterglow extends from 14 s (Eu^2+^,Nd^3+^) to 34 s with Pr^3+^, consistent with deeper/more numerous traps at 300 K. Printed dual-lifetime inks enable time-gated authentication using a binary 3 × 3 grid with two non-overlapping readout windows, illustrating a print-compatible blue/cyan security channel.

Although this study has made significant progress in the synthesis and application of BaAl_2_O_4_-based long afterglow materials, further optimization of the trap structure and stability of the materials is needed. Future research will focus on further improving the afterglow performance and exploring more doping combinations to meet a wider range of application requirements.

## Figures and Tables

**Table 1:** Summary of the stepwise optimization procedure for doping concentrations of Eu^2+^, Nd^3+^, and Pr^3+^ in BaAl_2_O_4_.

Step	Ion	Concentration Range (x, y, z)	Optimization Approach
Step 1	Eu^2+^	x = 0, 0.01, 0.02, 0.03, 0.04, 0.05	Optimized for maximum photoluminescence
Step 2	Nd^3+^	y = 0.01, 0.02, 0.03, 0.04, 0.05	Optimized for afterglow and emission properties
Step 3	Pr^3+^	z = 0.0005, 0.001, 0.0015, 0.0020, 0.0025	Optimized for further enhancing afterglow performance
Temperature Range	Eu^2+^, Nd^3+^, Pr^3+^	500–900 °C	After determining the optimal doping concentration, use this temperature range for sample synthesis at the optimum temperature.

**Table 2:** XRD data of BaAl_2_O_4_: Eu^2+^, Nd^3+^, Pr^3+^ phosphors.

2θ (°)	hkl	Intensity (I) (a.u.)	FWHM (β) (°)	Lattice Spacing (d) (Å)	Crystallite Size (D) (nm)	Dislocation Density (δ) (m^−2^)	Micro Strain (ε) (Dimensionless)
19.602	200	45	0.191	0.453	41.740	0.574	4.824
28.282	202	100	0.180	0.315	45.008	0.494	3.117
34.317	220	40	0.338	0.261	24.325	1.690	4.777
40.115	222	25	0.277	0.225	30.192	1.097	3.310
45.042	402	19	0.315	0.201	26.999	1.372	3.315

**Table 3:** Urbach-tail quantification.

Composition	E_U_ (eV)	95% CI (eV)	Fit Window (eV)	Points (*n*)	R^2^	S = kT/E_U_ (300 K)
BaAl_2_O_4_: Eu^2+^	0.656	0.643–0.669	3.71–4.01	25	0.9977	0.0394
BaAl_2_O_4_: Eu^2+^, Nd^3+^	0.38	0.373–0.386	2.85–3.15	42	0.997	0.0681
BaAl_2_O_4_: Eu^2+^, Nd^3+^, Pr^3+^	0.491	0.485–0.497	2.79–3.09	43	0.9984	0.0527

**Table 4:** Average fluorescence lifetimes of BaAl_2_O_4_: Eu^2+^, BaAl_2_O_4_: Eu^2+^, Nd^3+^, and BaAl_2_O_4_: Eu^2+^, Nd^3+^, Pr^3+^ phosphors.

BaAl_2_O_4_	Decay Lifetimes (μs)				
	A_1_	τ_1_	A_2_	τ_2_	τ*
Eu^2+^	16,847	60	73	712	92
Eu^2+^, Nd^3+^	15,046	10	32	480	54
Eu^2+^, Nd^3+^, Pr^3+^	15,880	10	10	500	25

Note: “τ*” denotes the average decay time calculated using Equation (6).

**Table 5:** Afterglow decay times of BaAl_2_O_4_: Eu^2+^, Nd^3+^ and BaAl_2_O_4_: Eu^2+^, Nd^3+^, Pr^3+^ phosphors.

BaAl_2_O_4_	Decay Lifetimes (s)				
	A_1_	τ_1_	A_2_	τ_2_	τ*
Eu^2+^, Nd^3+^	2159	2	126	29	14
Eu^2+^, Nd^3+^, Pr^3+^	181	2	25	44	34

Note: “τ*” denotes the average decay time calculated using Equation (6).

**Table 6:** Comparison of fluorescence lifetimes and afterglow decay times for BaAl_2_O_4_-based phosphors and SrAl_2_O_4_-based phosphors.

Material	Doping Concentration	Fluorescence Lifetime (μs/ns)	Afterglow Decay Time (s)
BaAl_2_O_4_: Eu^2+^	Eu^2+^ = 0.03	92 μs	0 s
BaAl_2_O_4_: Eu^2+^, Nd^3+^	Eu^2+^ = 0.03, Nd^3+^ = 0.03	54 μs	14 s
BaAl_2_O_4_: Eu^2+^, Nd^3+^, Pr^3+^	Eu^2+^ = 0.03, Nd^3+^ = 0.03, Pr^3+^ = 0.0015	25 μs	34 s
SrAl_2_O_4_: Eu^2+^	Eu^2+^ = 0.02	404 ns	0 s
SrAl_2_O_4_: Eu^2+^, Nd^3+^	Eu^2+^ = 0.02, Nd^3+^ = 0.01	46 ns	13 s

**Table 7:** Comparative summary of aluminate persistent phosphors for printing/security.

Host System	Representative Activators/Co-Dopants	Typical Synthesis Route & T	Dominant Emission (Qualitative)	Persistence Window (Qualitative)	Ink/Printing Compatibility	Application Notes/Positioning
BaAl_2_O_4_	Eu^2+^ (emitter); Nd^3+^/Pr^3+^ (trap engineering)	Combustion (this work): ~600 °C; also solid-state reported	Cyan/blue (Eu^2+^ 5d–4f)	Second scale (~14 s → ~34 s via co-doping)	High (fine powders, low-T processing, screen-print inks)	Time-gated; print-ready
SrAl_2_O_4_	Eu^2+^ (emitter); Dy^3+^ (traps)	Solid-state, typically > 1200 °C; flux/atmosphere tuning common	Green (~520–530 nm)	Minutes–hours under optimized conditions	Moderate (higher-T process;)	Long-duration; signage
CaAl_2_O_4_	Eu^2+^ (emitter); Nd^3+^/Dy^3+^ (traps)	Solid-state/combustion (composition- dependent)	Blue (~440–460 nm)	Minute-class in literature (composition- dependent)	Moderate (depends on particle)	Blue; tunable

## Data Availability

Data is contained within the article.
